# Cardiac arrest while using the toilet: not uncommon and associated with adverse resuscitation profile

**DOI:** 10.1016/j.resplu.2025.101047

**Published:** 2025-08-05

**Authors:** Elizabeth D. Paratz, Carl Johann Hansen, Andre La Gerche, Dion Stub, Ziad Nehme, Ashanti Dantanarayana, Kelila Freedman, Andreas Pflaumer, Jodie Ingles, Bo Gregers Winkel, Jacob Tfelt-Hansen

**Affiliations:** a Department of Cardiology, St Vincent’s Hospital Melbourne, 41 Victoria Parade, Fitzroy, VIC 3065, Australia; b St Vincent’s Institute of Medical Research, 9 Princes St, Fitzroy, VIC 3065, Australia; c Faculty of Medicine, Dentistry & Health Sciences, The University of Melbourne, Grattan St, Parkville, VIC 3000, Australia; d Heart, Exercise & Research Lab, Victor Chang Cardiac Research Institute, 405 Liverpool St, Darlinghurst, NSW 2010, Australia; e Department of Cardiology, Alfred Hospital, 55 Commercial Rd, Prahran, VIC 3181, Australia; f The Heart Centre, Copenhagen University Hospital-Rigshospitalet, Blegdamsvej 9, Copenhagen 2100 Denmark; g Department of Forensic Medicine (Retsmedicinsk Institut), University of Copenhagen, Frederik V’s Vej 11, Copenhagen 2100, Denmark; h School of Public Health and Preventive Medicine, Monash University, 553 St Kilda Rd, Melbourne, VIC 3004, Australia; i Centre for Research & Evaluation, Ambulance Victoria, 31 Joseph St, Blackburn North, VIC 3130, Australia; j Department of Cardiology, Royal Children’s Hospital, 50 Flemington Rd, Parkville, VIC 3052, Australia; k Murdoch Children’s Research Institute, 50 Flemington Rd, Parkville, VIC 3052, Australia; l Genomics and Inherited Disease Program, Garvan Institute of Medical Research, and UNSW Sydney, 384 Victoria St, Darlinghurst, NSW 2010, Australia; m School of Clinical Medicine, Faculty of Medicine and Health, UNSW Sydney, Australia

**Keywords:** Mortality, Cardiac arrest, Resuscitation, Forensic medicine

## Abstract

**Background:**

Out-of-hospital cardiac arrest (OHCA) on the toilet has been reported to be common and possibly driven by straining or vagal stimulus. Toilet-associated OHCA may also create a challenging resuscitation environment.

**Methods:**

The national Danish sudden death registry and state-wide Australian End Unexplained Cardiac Death (EndUCD) registry were examined**.** Persons with a fatal OHCA aged 5–50 years with autopsy-confirmed cardiac or unascertained aetiology were included. Resuscitation-related, aetiological and forensic factors were compared between persons experiencing fatal toilet-associated OHCA versus elsewhere. A composite variable of physiological conditions creating pressure-load or pressure-sensitivity was created, comprising hypertrophic cardiomyopathy, aortic stenosis/coarctation, and aortic aneurysm/dissection.

**Results:**

Of 2,463young persons, 75 (3.0 %) experienced toilet-associated fatal OHCA while 2,388 (97.0 %) experienced out-of-toilet OHCA. Australians experienced toilet-associated OHCA 1.7 times more frequently than Danes (4.1 % vs 2.4 %, *p* = 0.016). Toilet-associated OHCA was less frequently witnessed (13.3 % vs 32.1 %, *p* = 0.001), with lower rates of bystander cardiopulmonary resuscitation (32.0 % vs 55.7 %, *p* < 0.0001) and shockable rhythm (5.9 % vs 23.8 %, *p* = 0.003) compared to non-toilet OHCA. Toxicological results were more frequently positive for illicit substances in toilet-associated OHCA (32.8 % vs 16.3 %, *p* < 0.0001). No differences were identified in OHCA aetiology, including rates of the composite variable of aetiologies such as hypertrophic cardiomyopathy and aortic dissection.

**Conclusion:**

3.0 % of young fatal OHCA of cardiac aetiology is toilet-associated, with almost double the rates of toilet-associated OHCA in Australia compared to Denmark. No differences in OHCA aetiology were identified in toilet-associated OHCA. Resuscitation-related factors were adverse in toilet-related OHCA, highlighting the need for innovative ways to recognise and respond to toilet-associated OHCA.

## Background

Out-of-hospital cardiac arrest (OHCA) is a major societal problem, affecting up to half a million young people each year^1^ and imposing large impacts on healthcare, society and the economy.^2^ Location of cardiac arrest and time-use analysis are increasingly appreciated as highly important in the optimization of cardiac arrest care as they may influence the subsequent success of the often-quoted ‘Chain of Survival’, in which each step to survival depends on the preceding link.^3^ To date, cardiac arrest during sporting activity or in public locations such as airports and casinos have attracted greatest visibility, particularly in terms of strategic defibrillator placement.^4^, ^5^, ^6^, ^7^, ^8^

OHCA on the toilet appears to be a not-uncommon event. In 1948, McGuire et al. highlighted the ‘notorious frequency of sudden and unexpected deaths of patients while using bed pans in hospitals’,^9^ while in 1990, Dr Berko Sikirov wrote that ‘probably every physician practising emergency medicine has encountered tragic cases of sudden death in the lavatory…therefore it is a routine practise in coronary care to administer laxatives or stool softeners, hopefully to reduce straining at defecation’.^10^

Hypotheses for the cause and frequency of toilet-associated OHCA include the possibility of elevated rates of OHCA in patients with aortic aneurysms or hypertrophic cardiomyopathy where straining may induce aneurysmal rupture due to high pressure or a dynamic left ventricular outflow tract gradient (the Valsalva manoeuvre).^11^ Alternatively, increased parasympathetic tone (the Bezold-Jarisch reflex) has been implicated in both the pathogenesis of OHCA (as a cause of either profound bradyarrhythmias or a precipitant of bradycardia-induced ventricular arrhythmias in long QT syndromes),^12^, ^13^ or in creating a sudden urge to enter the toilet environment for excretory purposes.^14^, ^15^ OHCA on the toilet may also create a unique environment in terms of extrication for resuscitation.

This study was designed as a bi-national study of two comprehensive OHCA registries of OHCA in young people with adjudicated causes of OHCA to identify contemporary rates, predictors and management of OHCA in the restroom.

## Methods

### Data sources

#### Danish cohort

Data were extracted from the national Danish registry (population 5.9 million) for sudden death for the years 2000–2019.^16^ In Denmark, all citizens are allocated a National Person Registry ID, utilised in all healthcare encounters.^17^ The national Danish sudden death registry review all cases of sudden death by a multisource approach, utilising death certificates, postmortem reports and ambulance data.^18^

#### Australian cohort

Data were extracted from the End Unexplained Cardiac Death (EndUCD) registry for the years 2019–2023.^19^ The EndUCD registry covers the population of the state of Victoria, Australia (population 6.5 million), combining ambulance data of all sudden cardiac arrests aged 1–50 years attended by Ambulance Victoria with forensic data (post-mortem reports, police narratives of circumstances and toxicology reports) obtained from the National Coronial Information System (NCIS).^20^

### Methodology

#### Inclusion criteria

Persons with a fatal OHCA aged 5–50 years who underwent autopsy with confirmed cardiac or unascertained aetiology of their sudden death were included in the study. Exclusion criteria were age <5 years old (as it was considered that this population would not necessarily use a toilet independently), emergency services-witnessed OHCA, non-cardiac cause of OHCA identified on post-mortem assessment, survival from OHCA, no autopsy performed or location of OHCA not stated in either autopsy or police reports.

Data from the two registries was combined. Patients were defined by location as belonging to one of two populations: toilet-associated or non-toilet-associated OHCAs. Location of OHCA was only defined as occurring on the toilet if explicitly so stated in post-mortem examination or police reports; OHCAs specified as in the general bathroom environment, bath or shower were defined as non-toilet-associated events.

#### Autopsy investigation

Autopsies were conducted according to international recommendations for the post-mortem investigation of sudden cardiac death in the young, including detailed examination of cardiac structures and histopathology, with toxicology conducted where indicated (Table 1).^21^ With regards to aetiology of OHCA, a variety of aetiologies were defined including coronary artery disease, valvular disease, hypertrophic cardiomyopathy, aortic dissection, non-ischaemic cardiomyopathy and unascertained cause of OHCA. A composite variable was also created called ‘pressure-loaded or pressure-sensitive physiology’, comprising conditions in which obstructive physiology or dynamic generation of increased intra-thoracic pressure might hypothetically create higher risks in the setting of straining on the toilet, comprising HCM, pulmonary hypertension, valvular disease, coarctation of the aorta and aortic aneurysm/dissection.

**Table 1:** Definitions used within the study.

	Inclusion	Comments
Cause of death		
Aortic dissection	Aortic dissection identified	
Hypertrophic cardiomyopathy	Presence of morphological/histological features of hypertrophic cardiomyopathy ie asymmetric septal hypertrophy, myocardial disarray	
Valvular disease	Aortic stenosis/regurgitation, mitral stenosis/regurgitation/malignant mitral valve prolapse, infective endocarditis	Valvular condition assigned as primary cause of OHCA
Other cardiomyopathy	Non-ischaemic dilated cardiomyopathy, restrictive cardiomyopathy, arrhythmogenic cardiomyopathy, sarcoidosis	
Coronary disease	Atherosclerotic coronary disease, spontaneous coronary artery dissection, Kawasaki disease, Takayasu arteritis, anomalous coronary artery	Coronary disease assigned as primary cause of OHCA
Unascertained	Negative post-mortem examination: structurally normal heart. Sudden unexplained death or sudden arrhythmic death syndrome.	
Other	Pulmonary hypertension, aortic coarctation, myocarditis, non-valvular congenital heart disease	
Pressure-loaded or pressure-sensitive physiology	Composite variable of ‘pressure-loaded or pressure-sensitive’ physiology: hypertrophic cardiomyopathy, aortic dissection, valvular disease, pulmonary hypertension, aortic coarctation	
Forensic findings		
Toxicology positive for illicit drugs	Amphetamine-type substances, cannabis, cocaine, non-prescription opioids (ie heroin), novel psychoactive substances	Substances or their metabolites present
Toxicology positive for psychiatric drugs	Anti-depressants, anxiolytics, anti-psychotics, mood stabilisers	

### Statistical analysis

Groups were compared according to whether they were a toilet or non-toilet-associated OHCA using a chi-squared test, Mann-Whitney test or paired *t*-test according to variable identity (categorical or continuous, with the normality of distribution assessed by Shapiro-Wilk testing). The significance threshold was set as a *p*-value of <0.05. In line with NCIS requirements, all cohorts reporting only Australian data with a cell frequency of <5 were reported as ‘<5’ to maintain confidentiality. All statistical analysis was performed using STATA 17.0 (STATACorp, Texas, USA).

### Ethical approval

The study was approved by the Data Protection Agency in Copenhagen (2024-52-0093). The EndUCD registry holds overarching ethical approval from Alfred Health Human Research Ethical Committee (Study 597/18) and approval from the Justice Human Research Ethics Committee (Project M0468) for the use of NCIS data. This study was designed to comply with both the STROBE (Strengthening the Reporting of Observational Studies in Epidemiology) guidelines for observational studies and with the Declaration of Helsinki.

## Results

2463 persons were included in the study (Australian = 922, Danish = 1541), with 75 (3.0 %) being toilet-associated OHCAs and 2388 (97.0 %) being non-toilet associated OHCAs (Central Illustration). A difference between nationalities was observed, with Australians having a toilet-associated OHCA 1.7 times more frequently than Danes (4.1 % vs 2.4 %, *p* = 0.016) (Table 1).

### Resuscitation-related factors

**Fig. 1:**
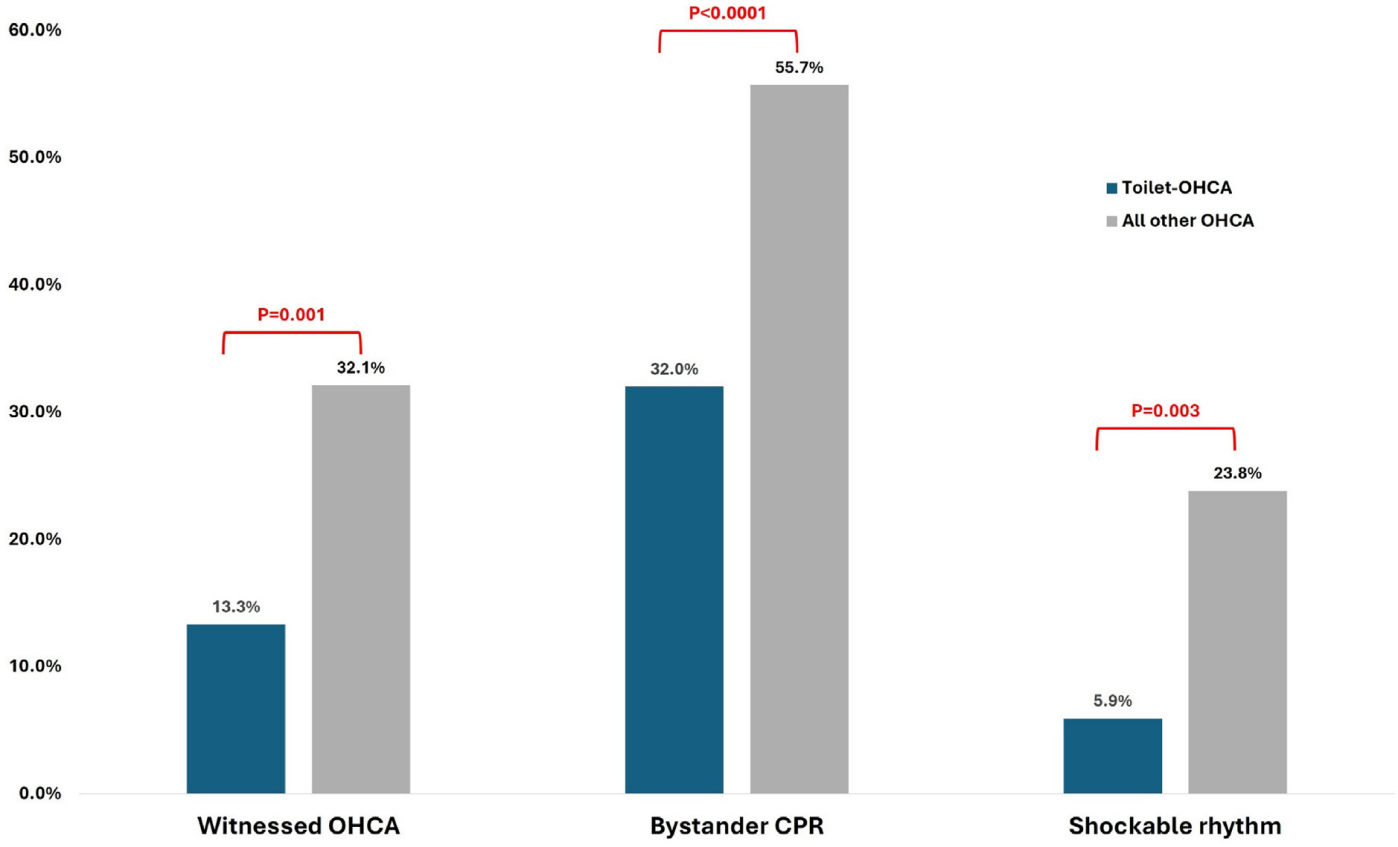
Unfavourable resuscitation-related factors were identified in the toilet-associated OHCA group. CPR = cardiopulmonary resuscitation; OHCA = out-of-hospital cardiac arrest.

Within the overall cohort, toilet-associated OHCAs were less likely to be witnessed than non-toilet-associated events (13.3 % vs 32.1 %, *p* = 0.001) (Fig. 1). Lower rates of bystander cardiopulmonary resuscitation (32.0 % vs 55.7 %, *p* < 0.0001) were also observed, with attendant lower rates of shockable rhythm (5.9 % vs 23.8 %, *p* = 0.003).

### Underlying aetiology of OHCA and forensic assessment

Toxicological results were more frequently positive for illicit substances in toilet-associated OHCA patients compared to non-toilet-associated patients (32.8 % vs 16.3 %, *p* < 0.0001). No differences were identified in the underlying cardiac cause of the OHCA, with leading causes of OHCA in both categories being coronary artery disease, non-ischaemic cardiomyopathy and unascertained cause of OHCA. There was similarly no difference in rates of the composite variable assessing obstructive physiology or complications of dynamically generating an increased pressure. No differences were also identified in median heart weight, rates of toxicological assessment or presence of psychiatric medications on toxicological assessments (Table 2).

**Table 2:** Clinical demographics, aetiology of OHCA and forensic findings.

	Toilet-associated OHCA (*n* = 75)	Non-toilet associated OHCA (*n* = 2388)	Significance
Demographics			
Age (years), median [IQR]	41 [33.2–46.2]	41 [32–46]	*P* = 0.2967
Male sex, *n* (%)	51 (68.0 %)	1761 (73.7 %)	*P* = 0.267
Body mass index (kg/m^2^)	27.8 [24.2–34.8]	27.8 [23.7–33.3]	*P* = 0.4357
Danish or Australian cohort, *n* (%)	Australian = 38 (50.7 %)	Australian = 884 (37.0 %)	***P* = 0.016**
Public location, *n* (%)	8 (10.7 %)	265 (11.1 %)	*P* = 0.846
Witnessed arrest, *n* (%)	10 (13.3 %)	753 (32.1 %)	***P* = 0.001**
Bystander CPR, *n* (%)	24 (32.0 %)	818 (55.7 %)	***P* < 0.0001**
Shockable rhythm, *n* (%)	3 (5.9 %)	349 (23.8 %)	***P* = 0.003**
Cause of OHCA			
Coronary disease, *n* (%)	23 (30.7 %)	832 (34.8 %)	*P* = 0.455
Aortic dissection, *n* (%)	4 (5.3 %)	107 (4.5 %)	*P* = 0.726
Hypertrophic cardiomyopathy, *n* (%)	0 (0.0 %)	24 (1.0 %)	*P* = 0.383
Valvular disease, *n* (%)	1 (1.3 %)	47 (2.0 %)	*P* = 0.695
Other cardiomyopathy, *n* (%)	16 (21.3 %)	487 (20.4 %)	*P* = 0.842
Unascertained, *n* (%)	23 (30.7 %)	733 (30.7 %)	*P* = 0.996
Pressure-loaded or pressure-sensitive physiology, *n* (%)*	6 (8.0 %)	183 (7.7 %)	*P* = 0.914
Forensic findings			
Heart weight (g), median[IQR]	450 [391–529]	442 [365–541]	*P* = 0.4115
Toxicology performed, *n* (%)	59 (78.7 %)	1737 (72.7 %)	*P* = 0.493
Toxicology positive for illicit drugs, *n* (%)	20 (32.8 %)	333 (16.3 %)	***P* < 0.0001**
Toxicology positive for psychiatric drugs, *n* (%)	5 (8.2 %)	139 (6.8 %)	*P* = 0.666

CPR = cardiopulmonary resuscitation; IQR = interquartile range; OHCA = out-of-hospital cardiac arrest.

* Composite variable comprising hypertrophic cardiomyopathy, aortic dissection, valvular disease, pulmonary hypertension or aortic coarctation.

## Discussion

**Fig. 2:**
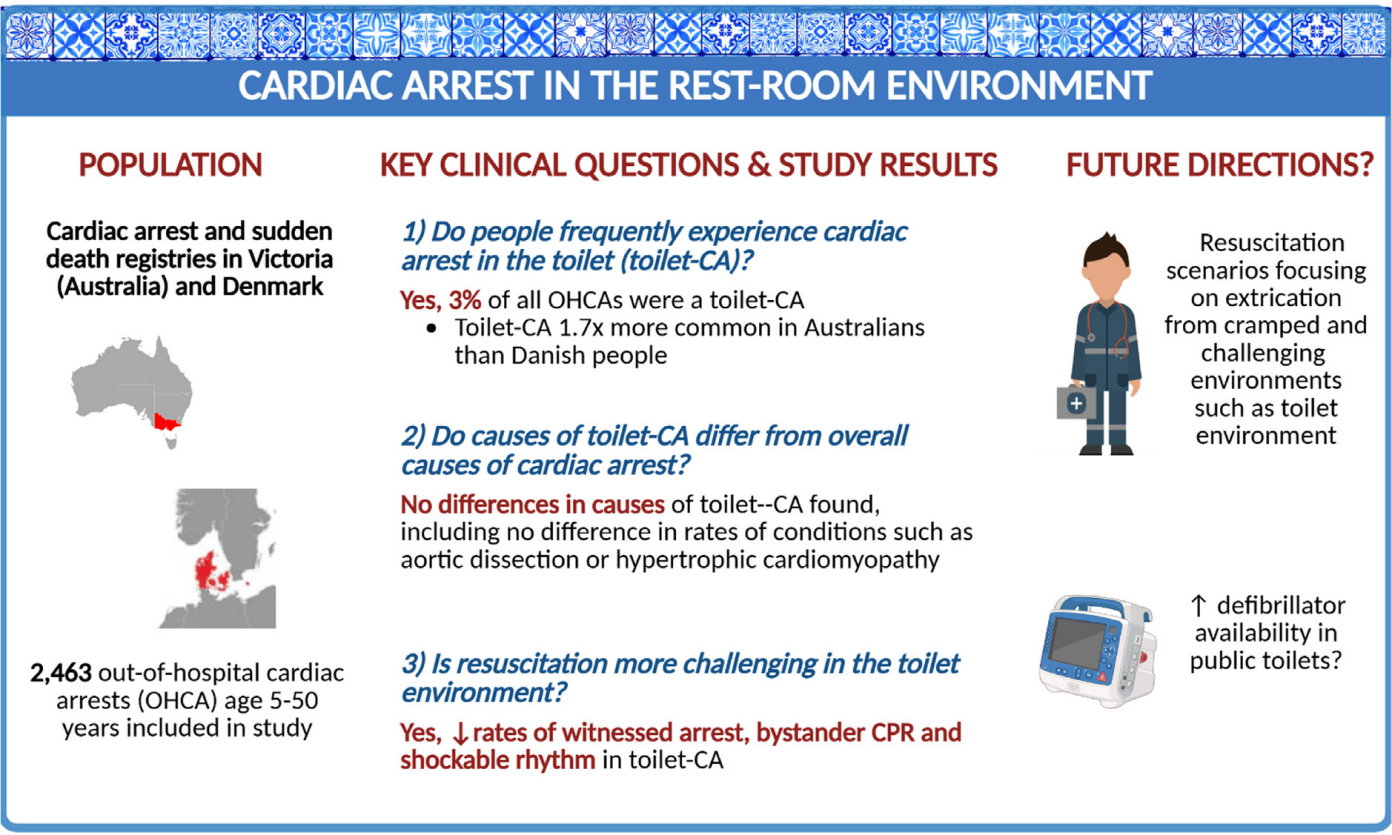
Central illustration. Overview of study design and findings. CA = cardiac arrest; CPR = cardiopulmonary resuscitation; OHCA = out-of-hospital cardiac arrest.

This study demonstrated that 3 % of fatal OHCA in young people occurs on the toilet, with almost double the rate of toilet-related cardiac arrest seen in the Australian cohort compared to the Danish. No significant differences in underlying aetiology of OHCA were identified in people who experienced OHCA on the toilet, including in rates of the composite variable. The major differences between groups lay in resuscitation-related factors, highlighting the need for consideration of unique environments in professional and bystander resuscitation training (Fig. 2).

### Our study in context

Japan has driven the majority of research into toilet-related OHCA.^22^ In the 1980s, Matoba et al. examined location of arrest in a cohort of 1230 sudden deaths undergoing autopsy in Osaka, with 8 % identified to have occurred on the toilet.^23^ In 1996, Hayashi et al. identified that rates of both non-fatal myocardial infarction and sudden cardiac death on the toilet significantly exceeded the estimated 2.1 % rate that would be expected from anticipated time spent on the toilet.^24^ A Japanese review of OHCAs in 2011 identified an 11 % rate of OHCAs occurring in the toilet with only a 1 % survival rate due to prolonged downtime prior to discovery. This cohort included non-cardiac causes of arrest, comprising 23 % of the cohort.^25^ An analysis by Moriwaki et al. in 2014 reported an 11.7 % rate of OHCA on the toilet.^26^ More recent Japanese studies by Kiyohara et al. in 2018 identified a 4.6 % and 4.8 % rate of toilet-associated OHCA respectively.^27^, ^28^

Studies elsewhere in the world include the United States of America; Fisher-Hubbard et al. analysed 2766 deaths referred to the Wayne County Medical Examiner’s Office in Michigan, USA and identified that 21 (0.8 %) occurred upon the toilet.^29^ This study was not limited to cardiac causes of death, and included cases of suicide, drug overdose and smoke inhalation. In Australia, a previous analysis of 725 patients aged 1–50 years experiencing OHCA (some of whom are included in this study) was linked to national time-use data, with toilet-OHCA rates reported at 8.0 % compared to national time-use data suggesting that all time spent in the toilet daily (including activities such as dental care and showering) would take only 3.4 % of the day.^20^ Overall, these studies suggest that rates of toilet-related OHCA range from 0.8 % to 11.7 %, with marked international variation.

High levels of international variation were seen within this study, with almost double the rates of toilet-related OHCA in Australia compared to Denmark. Calculation of ‘dose-exposure time’ in terms of time spent on toilet was challenging, due to limited data available according to age, sex and nationality, however a rough estimate of dose-exposure time is important to identify whether OHCA on-toilet occurs disproportionately frequently. ‘Smartphone lavatory syndrome’ has been described as the phenomenon of younger people (such as the 5–50 year age group included in our study) spending prolonged periods of time on the toilet engaged with their mobile phone^30^ and it would be valuable to have specific time-use data in this age group. National differences in time spent in the toilet have been described,^31^, ^32^ and a leading Danish toilet manufacturer has reported that the typical Dane spends 15 min per day on-toilet; however comparable Australian statistics are unavailable.^33^ Beyond national differences, even sex-related differences have been identified in toilet usage, with men typically spending 1.7 times as long on the toilet as women, further confounding attempts at dose-exposure calculation within this study.^34^

Another potential explanation for the difference seen between the two cohorts is the different time-periods covered, with the Australian cohort’s data substantially overlapping with the COVID-19 pandemic. The state of Victoria in Australia had strict COVID-19 lockdown orders, enforced with five-kilometre radius boundaries and nocturnal curfews.^35^ It is possible that relatively increased time was spent in the toilet in the Victorian cohort during the COVID-19 pandemic, explaining the higher proportion of toilet-OHCAs seen in the Australian cohort.

### Reasons underlying OHCA in the toilet environment

It has been proposed that patients feeling unwell may retreat to the bathroom environment^15^, ^25^, ^27^ – either due to concern regarding urgent bodily functions or simply for privacy. Likewise, it has been hypothesised that the act of straining to open bowels with a Valsalva manoeuvre may increase the risk of sudden cardiac arrest, particularly in the setting of obstructive physiology (ie aortic stenosis or hypertrophic cardiomyopathy) or aortic fragility vulnerable to dissection.^29^, ^36^ Of note, in McGuire’s original 1948 study, four of the 11 ‘bed-pan deaths’ had syphilitic aortitis, and another two had alternative aortic pathology.^9^ This longstanding hypothesis of toilet deaths being over-represented in certain aetiologies of OHCA did not appear to be substantiated within our study, including within the composite variable who had conditions previously reported to be high-risk for toilet-related OHCA. The higher rate of non-shockable rhythms in the toilet-associated OHCA group may relate to higher rates of bradyarrhythmias (as described in the Bezold-Jarisch reflex)^37^ or be simply a function of lower rates of witnessed OHCA.

We did observe higher rates of illicit substances detected on toxicology in the toilet-associated OHCA group, which may relate to illicit substances often being used in the toilet environment. However, these deaths were not caused by illicit drug overdose but to a cardiovascular cause with positive illicit toxicology as part of the post-mortem, therefore it seems unlikely that all patients had taken drugs immediately prior to collapse. It would be of interest to explore this finding further.

### Resuscitation in the toilet environment

It has been consistently reported that patients experiencing OHCA on the toilet experience worse survival and neurologic outcomes; this is thought to be predominantly driven by high rates of unwitnessed OHCA.^15^, ^26^, ^27^ Additional complicating factors with toilet-related resuscitations may include locked doors, biohazard exposure for resuscitation staff and a cramped ergonomically-challenging environment presenting extrication difficulties.^15^

From the findings of this study, we would make two major recommendations given the frequency of OHCA occurring in the toilet. Firstly, consideration could be given to widespread defibrillator placement in proximity of public toilets. Many initiatives have driven the placement of defibrillators in sporting areas on the basis that OHCA during exertion is common.^6^ Our data suggests that, with a prevalence of 3.0 %, toilet-associated OHCA may be common enough to warrant focussed initiatives to improve resuscitation outcomes.

Secondly, resuscitation in the toilet environment should be practised by ambulance personnel and at cardiopulmonary resuscitation courses. We would propose that training for unusual scenarios such as the toilet environment should be a specific simulated scenario in resuscitation education.

#### Limitations

This study includes only people aged 5–50, whose toilet habits may differ from the broader general population; for example some studies have suggested they may spend more time in the toilet environment. Data relating to pre-existing comorbidities and medications would be of high interest; for example diuretic prescriptions would be valuable to compare in the toilet versus non-toilet-associated OHCA populations. Some data variables were unavailable across both cohorts, and it would be interesting to link broader datasets capturing full ambulance-related information and medical history. Within the composite variable, some assumptions were made; for example, hypertrophic cardiomyopathy was assumed to be obstructive phenotype in all cases although this is unlikely to have been the case. Finally, this study included only deceased individuals, and so does not capture any variation that might have been present within the sudden cardiac arrest survivor cohort.

## Conclusion

This study identified that 3.0 % of OHCA in the young occurs while in the toilet environment, with almost double the rates of toilet-related OHCA seen in Australia compared to Denmark. No significant differences in underlying aetiology of OHCA were identified between people who were experienced OHCA on or off the toilet, including in rates of the composite variable. Resuscitation-related factors were adverse in people who experienced OHCA on the toilet, highlighting the need for innovative ways to recognise and respond to toilet-associated OHCA.

## Funding sources

EDP is supported by a Mamoma Foundation Fellowship, Heart Foundation Vanguard Grant, Sylvia & Charles Viertel Clinical Investigator Grant and Wilma Beswick Fellowship from the 10.13039/501100001782University of Melbourne. ALG is supported by an NHF Future Leadership Fellowship and NHMRC Investigator Grant. DS is supported by an NHF Future Leadership Fellowship and NHRMC Investigator grant. ZN is supported by an NHF Future Leadership Fellowship and NHMRC Investigator Grant. JI is the recipient of a National Heart Foundation of Australia Future Leader Fellowship and NHMRC Investigator Grant.

## CRediT authorship contribution statement

**Elizabeth D. Paratz:** Conceptualization, Data curation, Methodology, Writing – original draft. **Carl Johann Hansen:** Conceptualization, Data curation, Methodology, Writing – review & editing. **Andre La Gerche:** Methodology, Supervision, Writing – review & editing. **Dion Stub:** Funding acquisition, Methodology, Supervision, Writing – review & editing. **Ziad Nehme:** Conceptualization, Project administration, Writing – review & editing. **Ashanti Dantanarayana:** Data curation, Project administration, Resources, Writing – review & editing. **Kelila Freedman:** Project administration, Resources, Software, Writing – review & editing. **Andreas Pflaumer:** Conceptualization, Methodology, Project administration, Writing – review & editing. **Jodie Ingles:** Data curation, Methodology, Resources, Writing – review & editing. **Bo Gregers Winkel:** Conceptualization, Data curation, Methodology, Project administration, Supervision, Writing – review & editing. **Jacob Tfelt-Hansen:** Conceptualization, Methodology, Supervision, Writing – review & editing.

## Declaration of competing interest

The authors declare that they have no known competing financial interests or personal relationships that could have appeared to influence the work reported in this paper.

## References

[1] Marijon E., Narayanan K., Smith K. (2023). The Lancet Commission to reduce the global burden of sudden cardiac death: a call for multidisciplinary action. Lancet.

[2] Paratz E.D., Smith K., Ball J. (2021). The economic impact of sudden cardiac arrest. Resuscitation.

[3] Semeraro F., Greif R., Bottiger B.W. (2021). European Resuscitation Council guidelines 2021: systems saving lives. Resuscitation.

[4] Karlsson L., Malta Hansen C., Wissenberg M. (2019). Automated external defibrillator accessibility is crucial for bystander defibrillation and survival: a registry-based study. Resuscitation.

[5] Hansen S.M., Hansen C.M., Folke F. (2017). Bystander defibrillation for out-of-hospital cardiac arrest in public vs residential locations. JAMA Cardiol.

[6] Paratz E., Page G.J., Jennings G.L. (2023). Defibrillator access across Australia: the first step in avoiding a chain of fatality. Med J Aust.

[7] Paratz E.D., La Gerche A. (2022). The flatlining of cardiac arrest survival: can we revive the upward trend?. Eur Heart J.

[8] Shekhar A.C., Ruskin K.J. (2022). Sudden cardiac arrest in commercial airports: Incidence, responses, and implications. Am J Emerg Med.

[9] McGuire J., Green R.S., Courter S. (1948). Bed pan deaths. Trans Am Clin Climatol Assoc.

[10] Sikirov B.A. (1990). Cardio-vascular events at defecation: are they unavoidable?. Med Hypotheses.

[11] Ishiyama Y., Hoshide S., Mizuno H., Kario K. (2019). Constipation-induced pressor effects as triggers for cardiovascular events. J Clin Hypertens (Greenwich).

[12] Mahanta D., Barik R., Budhia A.K., Das D., Acharya D. (2023). A case of bradycardia-induced torsades de pointes in a patient presenting to the emergency room with cardiac arrest. Cureus.

[13] Sampson C.S., Shea C.M. (2016). Death by disimpaction: a bradycardic arrest secondary to rectal manipulation. Case Rep Emerg Med.

[14] Kumar A., Goyal A., Rehman F., Hariharan U. (2022). Vagal stimulation causing intra-operative cardiac arrest, a major dilemma whether to proceed or to defer surgery: a case report. Explorat Res Hypoth Med.

[15] Bates J. (2017). Toilets are no place for resuscitating patients. Nurs Stand.

[16] Hansen C.J., Svane J., Warming P.E. (2025). Declining trend of sudden cardiac death in younger individuals: a 20-year nationwide study. Circulation.

[17] Winkel B.G., Holst A.G., Theilade J. (2011). Nationwide study of sudden cardiac death in persons aged 1–35 years. Eur Heart J.

[18] Hansen C.J., Svane J., Palsoe M.K. (2023). Toxicology screening in sports-related sudden cardiac death: a multinational observational study. JACC Clin Electrophysiol.

[19] Paratz E.D., Rowsell L., van Heusden A. (2021). The End Unexplained Cardiac Death (EndUCD) registry for Young Australian Sudden Cardiac Arrest. Heart Lung Circ.

[20] Paratz E.D., van Heusden A., Zentner D. (2022). Causes, circumstances, and potential preventability of cardiac arrest in the young: insights from a state-wide clinical and forensic registry. Europace.

[21] Kelly K.L., Lin P.T., Basso C. (2022). Sudden cardiac death in the young: a consensus statement on recommended practices for cardiac examination by pathologists from the Society for Cardiovascular Pathology. Cardiovasc Pathol.

[22] Tokyo Toilet Tokyo, Japan; 2024 [available from: https://tokyotoilet.jp/en/].

[23] Matoba R., Shikata I., Iwai K. (1989). An epidemiologic and histopathological study of sudden cardiac death in Osaka Medical Examiner's Office. Jpn Circ J.

[24] Hayashi S., Toyoshima H., Tanabe N. (1996). Activity immediately before the onset of non-fatal myocardial infarction and sudden cardiac death. Jpn Circ J.

[25] Inamasu J., Miyatake S. (2013). Cardiac arrest in the toilet: clinical characteristics and resuscitation profiles. Environ Health Prev Med.

[26] Moriwaki Y., Tahara Y., Iwashita M., Kosuge T., Suzuki N. (2014). Risky locations for out-of-hospital cardiopulmonary arrest in a typical urban city. J Emerg Trauma Shock.

[27] Kiyohara K., Nishiyama C., Kiguchi T., Kobayashi D., Iwami T., Kitamura T. (2018). Out-of-hospital cardiac arrests in the toilet in Japan: a population-based descriptive study. Acute Med Surg.

[28] Kiyohara K., Nishiyama C., Matsuyama T. (2019). Out-of-hospital cardiac arrest at home in Japan. Am J Cardiol.

[29] Fisher-Hubbard A.O., Kesha K., Diaz F., Njiwaji C., Chi P., Schmidt C.J. (2016). Commode cardia-death by Valsalva maneuver: a case series. J Forensic Sci.

[30] Berney C.R. (2020). Smartphone lavatory syndrome. ANZ J Surg.

[31] Goldstein O., Shaham Y., Naftali T., Konikoff F., Lavy A., Shaoul R. (2009). Toilet reading habits in Israeli adults. Neurogastroenterol Motil.

[32] Gwynne S.M.V., Hunt A.L.E., Thomas J.R., Thompson A.J.L., Séguin L. (2019). The toilet paper: bathroom dwell time observations at an airport. J Build Eng.

[33] DB K. Vi nyder vores alene-tid på toilettet; 2014 [available from: https://www.dr.dk/levnu/krop/vi-nyder-vores-alene-tid-paa-toilettet].

[34] Bathstore. Someone's figured out how much of your life you're wasting on the toilet triple M; 2018 [available from: https://www.triplem.com.au/story/someones-figured-out-how-much-of-your-life-youre-wasting-on-the-toilet-92345].

[35] L M. (2021). Reflections on one of the world’s harshest COVID-19 lockdowns, and on the possibility of eliminating COVID-19 in Australia. HPHR.

[36] Inamasu J., Tomiyasu K., Miyatake S., Mayanagi K., Yoshii M., Nakatsukasa M. (2018). Clinical characteristics of stroke occurring in the toilet: are older adults more vulnerable?. Geriatr Gerontol Int.

[37] Al M. (1983). The Bezold-Jarisch reflex revisited: clinical implications of inhibitory reflexes originating in the heart. J Am Coll Cardiol.

